# COVID-19 Secondary Infections in ICU Patients and Prevention Control Measures: A Preliminary Prospective Multicenter Study

**DOI:** 10.3390/antibiotics11081016

**Published:** 2022-07-28

**Authors:** Sergio Ruiz-Santana, María-Luisa Mora-Quintero, Pedro Saavedra, Raquel Montiel-González, Catalina Sánchez-Ramírez, Guillermo Pérez-Acosta, Mar Martín-Velasco, Cristóbal Rodríguez-Mata, José-Manuel Lorenzo-García, Dácil Parrilla-Toribio, Tanya Carrillo-García, Juan-Carlos Martín-González

**Affiliations:** 1 Intensive Care Unit, Hospital Universitario Dr. Negrín, University of Las Palmas de Gran Canaria, Barranco de la Ballena s/n, E-35010 Las Palmas de Gran Canaria, Spain; catalinasanchezramirez@gmail.com (C.S.-R.); cristobal.rodriguez.mata@outlook.com (C.R.-M.); 2 Intensive Care Unit, Hospital Universitario de Canarias (Tenerife), Carretera de Ofra s/n, E-38320 San Cristóbal de La Laguna, Spain; mmorquic@gobiernodecanarias.org (M.-L.M.-Q.); jlorgarg@gobiernodecanarias.org (J.-M.L.-G.); 3 Department of Mathematics, University of Las Palmas de Gran Canaria, E-35010 Las Palmas de Gran Canaria, Spain; pedro.saavedra@ulpgc.es; 4 Intensive Care Unit, Hospital Universitario La Candelaria, Carretera General del Rosario 145, E-38010 Santa Cruz de Tenerife, Spain; raquelmontiel@gmail.com (R.M.-G.); mar.martinvelasco@gmail.com (M.M.-V.); dpartor@gmail.com (D.P.-T.); 5 Intensive Care Unit, Complejo Hospitalario Universitario Insular-Materno Infantil, Avenida Marítima del Sur s/n, E-35016 Las Palmas de Gran Canaria, Spain; guillermoastinor@gmail.com (G.P.-A.); tanya.carrillo93@gmail.com (T.C.-G.); jmargonn@gobiernodecanarias.org (J.-C.M.-G.)

**Keywords:** SARS-CoV-2, COVID-19, infection control, decontamination, drug resistance, bacterial, pneumonia, ventilator-associated

## Abstract

The incidence of secondary infections in critically ill coronavirus disease 2019 (COVID-19) patients is worrisome. We investigated whether selective digestive decontamination (SDD) added to infection control measures during an intensive care unit (ICU) stay modified these infection rates. Methods: A retrospective observational cohort study was carried out in four ICUs in Spain. All consecutive ventilated patients with a SARS-CoV-2 infection engaged in national infection control programs between 1 March and 10 December 2020 were investigated. Patients were grouped into two cohorts according to the site of ICU admission. Secondary relevant infections were included. Infection densities corresponding to ventilator-associated pneumonia (VAP), catheter bacteremia, secondary bacteremia, and multi-resistant germs were obtained as the number of events per 1000 days of exposure and were compared between SDD and non-SDD groups using Poisson regression. Factors that had an independent association with mortality were identified using multidimensional logistic analysis. Results: There were 108 patients in the SDD cohort and 157 in the non-SDD cohort. Patients in the SDD cohort showed significantly lower rates (*p* < 0.001) of VAP (1.9 vs. 9.3 events per 1000 ventilation days) and MDR infections (0.57 vs. 2.28 events per 1000 ICU days) and a non-significant reduction in secondary bacteremia (0.6 vs. 1.41 events per 1000 ICU days) compared with those in the non-SDD cohort. Infections caused by MDR pathogens occurred in 5 patients in the SDD cohort and 21 patients in the non-SDD cohort (*p =* 0.006). Differences in mortality according to SDD were not found. Conclusion: The implementation of SDD in infection control programs significantly reduced the incidence of VAP and MDR infections in critically ill SARS-CoV-2 infected patients.

## 1. Introduction

The initial symptoms of coronavirus disease 2019 (COVID-19) usually affect the respiratory and gastrointestinal systems. The development of dyspnea linked to hypoxemia results from activation of the host inflammatory pathways. Many patients with severe disease, particularly those who are unvaccinated or immunosuppressed, present viral severe acute respiratory distress syndrome (ARDS), with lung bilateral infiltrates and severe hypoxemia. This severe lung infection has a role in the excessive proinflammatory cell recruitment and cytokine release, which contribute to alveolar and full-body endothelial damage. Cardiovascular complications may also develop, including direct virus myocarditis and myocardial infarction that may lead to cardiogenic shock. Acute kidney and liver injury, together with rhabdomyolysis, coagulopathy, and distributive shock, are among the salient extrapulmonary manifestations of COVID-19. These organ failures may be associated with clinical and laboratory signs of inflammation, including fever, thrombocytopenia, hyperferritinemia, and elevations of C-reactive protein and interleukin-6 (IL-6) [1]. Finally, COVID-19 causes disabilities as a result of post-intensive care syndrome and long COVID [2].

At the beginning of the pandemic, when patients were admitted to the hospital with respiratory failure due to SARS-CoV-2 infection, they were treated according to the best available evidence with a combination of antiviral treatments and antibiotics. Treatments usually included a combination of lopinavir/ritonavir and chloroquine/hydroxychloroquine, whereas antibiotic treatment was started usually at hospital admission with ceftriaxone and azithromycin. Corticosteroids were given to patients that required at least high flow oxygen, and nonresponding ARDS patients in the intensive care unit (ICU) commonly received a high dose of methylprednisolone. Tocilizumab was also given to those patients receiving non-invasive ventilation to block the IL-6 pathway [3].

Later in the pandemic, the RECOVERY trial changed the clinical practice and established the use of dexamethasone, particularly in those patients on mechanical ventilation [4]. Finally, the REMAP-CAP study showed that tocilizumab or sarilumab could further reduce mortality and organ-support-free days when started within 24 h of ICU admission [5]. The use of remdesivir to prevent the progression to severe COVID-19 was also established and it was shown that this produced a faster recovery and lower use of supportive therapy in hospitalized patients [6,7]. In addition, we then learned that chloroquine/hydroxychloroquine alone or with azithromycin did not improve the clinical status at 15 days in mild-to-moderate COVID-19 as compared with standard care [8].

In critically ill COVID-19 patients, the appearance of bacterial, fungal, and viral secondary infections complicate their clinical course. The main COVID-19 secondary infections include ventilator-associated pneumonia (VAP) and systemic infections, such as bloodstream infection, COVID-19-associated pulmonary aspergillosis (CAPA), and invasive candidiasis [9,10]. Gram-negative bacteria, such as Enterobacterales and *Staphylococcus aureus*, with a notorious rate of multi-drug resistant (MDR) isolates, caused most of the cases of VAP [11]. A large percentage (17–32%) of patients hospitalized for SARS-CoV-2 infection require admission to the ICU, and about 10% of these patients require mechanical ventilation despite the use of high-flow nasal oxygen, noninvasive mechanical ventilation, and a prone position [12]. However, only 1% of them who were discharged alive had died before a 1-year follow-up [13].

The increase in secondary infections in COVID-19 patients admitted to the ICU is a matter of concern and the true incidence remains elusive [14], but it is high. A recent review of the literature showed that the incidence of MDR bacterial infections in critically ill COVID-19 patients is also high, ranging between 32% and 50%, with invasive mechanical ventilation, steroid therapy, and length of ICU stay as predisposing factors [15]. Infections caused by MDR pathogens may lead to acute respiratory distress syndrome (ARDS), multiorgan failure, prolonged mechanical ventilation, renal replacement therapy, or extracorporeal membrane oxygenation (ECMO) [16,17]. Moreover, corticosteroid therapy was shown to reduce mortality but its impact on secondary infections is not very well defined [18,19]. Other drugs, such as tocilizumab, may also lead to serious secondary infections [20].

Selective digestive decontamination (SDD) is a prophylactic treatment for critically ill patients that is based on an oropharyngeal paste and enteral suspension containing antimicrobials, usually tobramycin, colistin, and an antifungal, as well as an intravenous antibiotic (usually a second-generation cephalosporin) that is administered during the first 4 days of ICU admission. The aim of SDD is to prevent or eradicate, if present, the oropharyngeal and intestinal abnormal carriage of potentially pathogenic microorganisms, such as aerobic Gram-negative bacilli, methicillin-sensitive or resistant *Staphylococcus aureus* (in the latter, vancomycin is added to the SDD regimen), and yeasts, in patients at risk for nosocomial infections. Once a patient has been successfully decolonized, the unaffected anaerobic flora would offer prevention against new colonization by potentially pathogenic microorganisms. There are four published notorious studies that found a significant reduction in ICU and hospital mortality when comparing SDD to a placebo or standard of care [21,22,23,24]. A recent meta-analysis seems to corroborate these findings in ICUs with low levels of antibiotic resistance [25].

SDD in ICU patients was shown to prevent severe infections [26,27] and reduce mortality [22,28,29] but the use of this prophylactic measure is still controversial, especially in ICU settings with a high prevalence of MDR microorganisms [30,31], because it may contribute to antimicrobial resistance [32,33]. In a previous study undertaken by our group, the long-term use of SDD was effective in reducing the rates of VAP, secondary bloodstream infection, and antibiotic consumption while decreasing colistin, tobramycin, and most of the antibiotic-resistant colonization rates in a mixed ICU with a high endemic level of MDR microorganisms [34]. However, although preliminary data were recently published [24,35], additional results regarding the benefits of SDD in critically ill COVID-19 patients are needed.

We sought to assess differences in the incidence of ICU-acquired secondary and MDR infections in critically ill mechanically ventilated COVID-19 patients that were routinely placed in well-established ICUs in Spanish national infection control programs according to the use or non-use of SDD based on the hypothesis that fewer secondary and MDR infections would be associated with the addition of SDD. This study was conducted in four ICUs in the Canary Islands, where the excess of deaths for 2020 was smaller than for mainland Spain [36,37], similar to the lower impact of the pandemic on other islands [38].

## 2. Results

The study population included 265 critically ill COVID-19 patients, with 108 in the SDD cohort and 157 in the non-SDD cohort. In relation to the types of SARS-CoV-2 in Spain, from March to June 2020, A lineages predominated over B lineages, with half of the sequences belonging to lineage A.2 and less than 10% to lineage A.5. Then, at the end of June 2020, the presence of B lineages increased to nearly 80% and the most successful lineage circulating was B.1.177 [39]. Characteristics of the patients are shown in Table 1. There were statistically significant differences between the two cohorts in some variables, including ferritin and D-dimer levels, treatment with anticoagulants, prone position, use of central venous catheters, and use of corticosteroids. As is also shown in Table 1, the median ICU length of stay was not significantly different between both studied cohorts (*p* = 0.24). However, there was a non-significant reduction in the duration of mechanical ventilation in the SDD cohort compared with the non-SDD cohort (*p* = 0.9). Finally, there was also a lower non-significant ICU and hospital mortality rate in the SDD cohort compared with the non-SDD cohort (*p* = 0.32 and *p* = 0.21, respectively).

As shown in Table 2, regarding the primary endpoint, critically ill COVID-19 patients in the SDD cohort showed significantly lower rates of VAP (1.9 vs. 9.3 events per 1000 ventilation days; *p* < 0.001) and MDR infection (0.57 vs. 2.28 events per 1000 ICU days; *p* < 0.001) compared with those in the non-SDD cohort. The rate of secondary bacteremia was non-significantly lower in the SDD cohort (0.57 vs. 1.41 events per 1000 ICU days; *p* = 0.087), and the rate of catheter-related bloodstream infection/bacteremia of unknown origin was similar in both cohorts.

As displayed in Table 3, infections caused by MDR pathogens occurred in 5 patients in the SDD cohort and 21 in the non-SDD cohort (*p =* 0.006). As shown in Table 3, *Pseudomonas aeruginosa* was the most common pathogen, followed by *Escherichia coli* and *Klebsiella pneumoniae*. In VAP and secondary bacteremia, *P. aeruginosa* was the most frequent causative microorganism, although in catheter-related bloodstream infection/primary bacteremia, different pathogens were isolated. No MDR pathogen isolations in secondary bacteremia were recorded in the SDD cohort (Table 3). Overall, *P. aeruginosa* was more frequently isolated in the non-SDD cohort than in the SDD cohort. The results of the antibiotic susceptibility testing for each MDR pathogen isolated in all study patients are shown in Table S1 of the Supplementary Materials.

Finally, as shown in Table 4, the risk factors for ICU mortality, after excluding one hospital that covered a SARS-CoV-2 inundated area, were age (OR 95% CI: 1.043 (1.013; 1.073)), Apache II score on ICU admission (OR 95% CI: 1.044 (1.995; 1.096)), and the last value of serum D-dimer (OR 95% CI: 1.390 (1.090; 1.771)).

## 3. Discussion

The incidence of secondary infections in COVID-19 patients who were admitted to two ICUs that applied SDD showed a rate of VAP per 1000 ventilation days, and importantly, the number of isolations of MDR pathogens that were significantly lower compared with patients admitted to two other ICUs that did not use SDD under comparable infection control and preventive measures. Secondary bacteremia was also lower in the SDD cohort, although non-significantly. A methodological aspect to be considered is the external validity of the study sample, which is supported by independent official statistics as the number of admissions to the participating ICUs was superimposable to all ICU admission by SARS-CoV-2 infection in the Canary Islands during the study period [39].

Recently, Luque-Paz et al. [24] compared two independent cohorts of ICU COVID-19 patients from two different centers, with one applying SDD (n = 77) and the other without SDD (n = 101). They also found a large decrease in VAP incidence in the SDD cohort compared with the non-SDD cohort (9 vs. 23 VAP per 1000 ventilation-days, respectively). Unlike our findings, this decrease was associated with a significant decrease in mortality. A Dutch observational single-center non-comparative study also reported similar lower VAP results [35]. In addition to this, our study also evaluated the relationship between SARS-CoV-2 infection and the incidence of relevant secondary and MDR infections in two critically ill cohorts that received or did not receive SDD on top of specifically designed measures of ICU infection prevention control programs. The present findings are clinically applicable, not only from the prophylactic perspective of potentially life-threatening secondary and MDR infections in critically ill COVID-19 patients but also because SDD is an effective and cost-saving measure [40,41] that can be easily implemented in daily practice after optimizing ICU infection prevention control programs.

Bacterial or fungal secondary infections in COVID-19 patients and their relationship with mortality have also been a relevant consideration since the start of the pandemic; although its true incidence remains to be determined, it is high [42,43]. These superinfections are frequently caused by MDR pathogens that take advantage of conditions usually present in these patients: ARDS, which is sometimes in need of ECMO; multi-organ failure; prolonged mechanical ventilation; renal replacement therapy; and the use of drugs, such as corticosteroids or tocilizumab. Benefits of early administration of cytokine inhibitors, such as tocilizumab, seem to be associated with prolonged survival in COVID-19 patients, and the RECOVERY trial provided evidence that treatment with dexamethasone reduced 28-day mortality [4,44].

In a European multicenter cohort study, the incidence of ventilator-associated lower respiratory tract infections was reported to be significantly higher in patients with SARS-CoV-2 infection as compared with patients with influenza pneumonia or no viral infection [12]. The most common bacteria isolated were Gram-negative bacilli (83.6%), mainly *P. aeruginosa*, followed by Gram-positive cocci (19.5%), mainly methicillin-sensible and resistant *S. aureus*. Furthermore, there was a notorious 23.3% of MDR isolates [12]. In an ICU Italian multicenter retrospective analysis that included 774 adult patients with severe COVID-19, the authors found that these patients were at high risk of hospital-acquired infections, in particular, VAPs and bloodstream infections caused by MDR microorganisms [45]. The most frequent infections were VAPs, with 26 per 1000 patient intubation-days, bloodstream infections with 11.7 per 1000 ICU patient-days, and catheter-related bloodstream infections with 4.7 per 1000 ICU patient-days [45]. The Gram-negative bacteria *Enterobacterales* and *S. aureus* caused 64% and 28% of cases of VAP, respectively. Hospital-acquired infections prolonged mechanical ventilation and hospitalization and, when complicated by septic shock, nearly doubled mortality [30]. In our study, patients that received SDD had a significant decrease in the rate of VAP per 1000 mechanical ventilation days compared with those that did not (RR: 0.204, 95% CI: 0.112–0.371), with *P. aeruginosa* also being the most common isolated bacteria but with a very significant reduction in the SDD cohort.

ICU-acquired bloodstream infections were reported to have an increased incidence in COVID-19 patients admitted to the ICU [46,47]. In a matched-case cohort study, COVID-19 increased the daily risk of developing ICU-acquired bloodstream infections with an HR of 4.5 (95% CI: 1.82–11.16; *p* = 0.001), with coagulase-negative *Staphylococci* being the microorganism most frequently identified among COVID-19 patients [47]. In our study, we observed a non-significantly higher rate of catheter-related bloodstream infection/bacteremia of unknown origin in the SDD vs. non-SDD cohort. This was probably due to the fact that SDD does not interfere with these types of bloodstream infections; however, patients in the SDD cohort showed a non-significant reduction in secondary bacteremias that can be affected by the SDD protocol.

ICU patients with severe COVID-19 also showed a high prevalence of systemic candidiasis, with *C. albicans*, *C. parapsilosis*, and *C. glabrata* as frequently recovered fungal isolates [10,48]. We did not observe any case of candidemia, neither in the SDD nor in the non-SDD cohort. The prophylactic use of nystatin may have contributed to these findings in the SDD cohort.

When considering MDR pathogens, critically ill COVID-19 patients also have an increased risk of nosocomial MDR infections with high mortality [11]. Another interesting finding of our study was the significantly lower rate of MDR infections in the SDD cohort, with no MDR pathogens isolated among patients with secondary bacteremia treated with SDD. It is of note that both studied cohorts displayed germ resistance to third- and fourth-generation cephalosporins and carbapenems but only one germ developed resistance to aminoglycosides in the SDD cohort (Table S1, Supplementary Materials). Considering that both study cohorts were well balanced regarding age, the severity of disease, ICU length of stay, and days of mechanical ventilation, but not in terms of the use of corticosteroids in the SDD cohort, we think that a biologically plausible preliminary explanation for the observed MDR germs reduction may have been the preventive effects of SDD.

Comorbidities contribute to COVID-19 prognosis in a relevant way. In a recent study carried out in Spain, the authors retrospectively analyzed the characteristics and in-hospital outcomes of all patients admitted with COVID-19 in eight university hospitals in Catalonia over 1 year (February 2020–February 2021) [13]. Among the patients’ clinical characteristics, the presence of the relevant comorbidities was considered. It was found that hypertension, diabetes, and cardiovascular disease were the leading comorbidities in the overall study sample and each of the investigated periods. The comparison of comorbidities revealed significant differences between the first three COVID-19 waves regarding the proportion of patients with diabetes, hypertension, cardiovascular disease, and chronic kidney disease at the time of hospital admission. Overall, the proportion of patients with a Charlson score ≥ 3 increased in the second and third waves. The percentage of individuals with obesity at admission increased in the third wave. The proportion of patients in the lowest socioeconomic level increased after the first wave [13]. In our study, we did not find statistically significant differences in body mass index between both studied groups.

In mechanically ventilated patients with severe COVID-19, per 1-year increase in age, the OR of 180-day mortality was 1.05, but interestingly, the use of SDD showed an OR of 0.59 [49]. Our ICU mortality rate of 24.5% was in the lower range, most probably because, with the exception of one of the hospitals, we were in a SARS-CoV-2 non-inundated area and throughout the sequential COVID-19 waves, this fact produced more favorable outcomes. After excluding the hospital that covered a SARS-CoV-2 inundated area, in the multivariate analysis, age, Apache II score on admission, and the natural logarithm of the last determination of D-dimer were independent predictors of ICU death. Older age and higher D-dimer were also identified as risk factors that were significantly associated with mortality in critically ill COVID-19 patients requiring mechanical ventilation [50].

This study had several limitations. First, in addition to being a retrospective study, it was not a clinical trial. Despite this, we took advantage of the fact that in our community, there were two hospitals with ICUs with several years of experience applying SDD and another two that did not use it. Second, we also are aware of the difficulties of diagnosing VAP in COVID-19 patients because both entities share similar diagnosis criteria. This was the main reason why two of us centrally assessed and adjudicated these diagnoses before performing the statistical analysis. Third, another limitation may be the fact that this multicenter study was in fact performed, as stated, in different centers, and thus, there might exist unavoidable confounding factors among them that influenced the risk of infection. However, all the participant hospitals used common national nosocomial infection prevention bundles that at least lowered the risk of infection.

## 4. Material and Methods

### 4.1. Study Design and Participants

This was a retrospective observational study, with all variables recorded prospectively, and was designed with the participation of the medical-surgical ICUs of the four largest acute-care tertiary hospitals in the Canary Islands (Spain).

All consecutive critically ill patients with SARS-CoV-2 infection confirmed using nucleic acid amplification tests (NAAT) admitted between 1 March and 10 December 2020 were included. A length of ICU stay of at least three days was required. Patients were divided into two ICU-based cohorts according to the use or non-use of SDD after ICU admission. In two 42- and 24-bed ICUs, SDD has been a routinely implemented measure when SDD was a highly recommended component of the VAP prevention bundle in the nationwide “Pneumonia Zero” program [51]. In the other two 32- and 30-bed ICUs, SDD was never used, although all four ICUs participated equally in several nationwide projects sponsored by the Spanish Society of Intensive Care Medicine and Coronary Units (Semicyuc) (such as “Bacteremia Zero”, “Pneumonia Zero”, “Resistance Zero”, “Urinary Tract Infections Zero”). The only criteria, after COVID-19 diagnosis and after an ICU stay of at least 3 days on mechanical ventilation, that was used to assign a patient to SDD or non-SDD was exclusively having been admitted to an ICU in which SDD or non-SDD strategies were systematically used. Moreover, universal prophylactic anticoagulation in all critically ill COVID-19 patients, mostly with intermediate doses, was a routine measure applied in all participating ICUs. All the VAP infection events were analyzed and adjudicated by two of us (C.S.-R., S.R.-S.) before performing the statistical analysis.

### 4.2. Study Procedures

The SDD protocol was previously reported [34]. Briefly, it includes the use of an oral paste and oral suspension containing colistin, tobramycin, and nystatin, together with systemic cefotaxime, during the first 4 days of SDD, and the use of vancomycin in methicillin-resistant *Staphylococcus aureus* (MRSA) carriers. SDD was started on the day of tracheal intubation and was maintained throughout the length of ICU stay until discharge.

Surveillance samples from the throat, tracheostomy, rectum, and pressure sores were collected on ICU admission and once weekly thereafter. Diagnostic samples from tracheal aspirates, peripheral blood, urine, or surgical wounds were obtained at the physician’s discretion. Antimicrobial susceptibility testing was performed with the VITEK-2 system (bioMérieux, Inc., Durham, NC, USA) [52], and the breakpoints were defined according to the European Committee on Antimicrobial Susceptibility Testing [53] guidelines. Infections caused by MDR pathogens included extended-spectrum β-lactamase (ESBL) producing *Enterobacteriacea spp.* resistant to ceftazidime and/or aminoglycosides and/or ciprofloxacin, carbapemenase producing *Enterobacteriacea spp*., *Pseudomonas aeruginosa* resistant to ceftazidime and/or aminoglycosides and/or ciprofloxacin and/or imipenem, MRSA, any strain of *Acinetobacter* spp. resistant to carbapenems, Gram-negative bacteria resistant to three or more antimicrobial families, *Clostridioides difficile*, and vancomycin-resistant *Enterococcus* spp. These definitions of MDR pathogens were those used in the ENVIN-HELICS registry (National Nosocomial Infection Surveillance Study–Hospitals in Europe Link for Infection Control through Surveillance), a nationwide ongoing multicenter data collection system in which invasive device-related infections in ICU patients were recorded [54].

ICU-acquired infection was defined as the isolation of a new strain that was not recovered in any of the samples taken during the first 48 h of admission. Secondary infections included VAP, central-line-associated bloodstream infection/bacteremia of unknown origin, secondary bacteremia, and infection caused by MDR pathogens. Criteria for the definition of these infections were those used in the ENVIN-HELICS registry [54].

### 4.3. Endpoints

The primary endpoint of the study was the incidence of ICU-acquired secondary and MDR infections in mechanically ventilated critically ill COVID-19 patients in the SDD and non-SDD cohorts. Secondary endpoints were the length of stay in the ICU, the ICU and hospital mortality rates in the SDD and non-SDD cohorts, and the risk factors for ICU mortality.

### 4.4. Statistical Analysis

#### 4.4.1. Subjects and Measurements

This was a prospective study that included 265 critically ill patients with COVID-19 that underwent mechanical ventilation and had stayed in intensive care units for at least three days.

#### 4.4.2. Univariate Statistical Analysis

Categorical variables were expressed as frequencies and percentages and continuous variables as mean and standard deviation (SD) when data followed a normal distribution or as a median and interquartile range (IQR = 25th–75th percentile) when the distribution departed from normality. The percentages were compared using the chi-square ($\chi^{2}$) test, the means using the *t*-test, and the medians using the Wilcoxon test for independent data.

#### 4.4.3. Incidences per 1000 Days of Exposure

For each infection considered (nosocomial pneumonia, catheter-related bacteremia, secondary bacteremia, and multi-resistant germs), the number of events (Nh) and the total number of days of exposure (days_h) were available for each hospital h. Then, we considered a random effects Poisson model [55] that assumed that N_h~Poisson(ν_h⋅μ_h), where ν_h were continuous, positive-valued, independent, and identically distributed random variables of mean one and variance τ (overdispersion) and

ln(μ_h) = ln(days_h) + α + β⋅SDD_h:     SDD_h = 0, 1

where for each hospital h, SDD_h took values of 1 or 0 according to use or not of SDD, respectively.

#### 4.4.4. Multivariate Logistic Regression

In order to identify the factors that maintained an independent association with death, a multivariate logistic regression analysis was performed. Age, sex, hospital, severity marker (Apache II score on admission), renal function biomarkers, and initial and final D-dimer values were entered into the analysis. The selection of variables based on the best subset regression and Akaike information criterion (AIC) was then performed [56]. The model was summarized as *p*-values (likelihood ratio test) and odds ratios, which were estimated by means of 95% confidence intervals.

Statistical significance was set at *p* < 0.05. Data were analyzed using the R package, version 3.6.1 [57].

## 5. Conclusions

In conclusion, our preliminary results showed that in SARS-CoV-2-infected patients, the implementation of SDD in well-established infection control programs significantly reduced the incidence of VAP and MDR infections, together with a non-significant reduction in the incidence of secondary bacteremia. Results of the currently ongoing SuDDICU study (Selective Decontamination of the Digestive Tract in ICU patients) (ClinicalTrials.gov NCT02389036), which is a multicenter cluster, crossover, randomized controlled trial of SDD plus standard of care as compared with standard of care alone in mechanically ventilated ICU patients, will provide conclusive data since one of the secondary outcomes was to assess changes in antibiotic resistance rates between study epochs (pre-trial, interperiod gap, and post-trial) within groups. With a recruitment target of 15,000 participants in Canada, the United Kingdom, and Australia, the study will be completed in December 2023. It may be expected that forthcoming strong evidence will provide support for the present preliminary findings.

## Figures and Tables

**Table 1:** Characteristics of the patients according to the SDD regimen.

Variables	Overall N = 265	Non-SDD N = 157	SDD N = 108	*p*-Value
Age (years)	63.6 ± 11.9	64.2 ± 11.6	62.6 ± 12.2	0.272
Sex (male)	162 (61.1)	95 (60.5)	67 (62.0)	0.802
Body mass index (kg/m^2^)	29 (26; 33)	28 (26; 33)	29 (26; 33)	0.910
Apache II score on admission	14 (11; 18.5)	14 (10; 18)	15 (11; 19.7)	0.155
Follow-up days	33 (21; 54)	32 (20; 53)	35 (22; 58)	0.394
ICU days	21 (12; 35)	20 (11; 35)	23 (14; 33)	0.247
Death ICU	75 (28.3)	48 (30.6)	27 (25.0)	0.322
Death, hospital	80 (30.2)	52 (33.1)	28 (25.9)	0.210
Deep venous thrombosis	6 (2.3)	3 (1.9)	3 (2.8)	0.690
PaO_2_/FIO_2_	133 (97; 200)	138 (98; 200)	129 (97; 191)	0.501
At ICU admission:				
Albumin (mg/dL)	2.90 (2.57; 3.20)	2.89 (2.52; 3.19)	3.00 (2.60; 3.20)	0.176
Urea (mg/dL)	45 (33; 62)	46 (33; 62)	44 (32; 63)	0.973
Creatinine (mg/dL)	0.89 (0.70; 1.18)	0.83 (0.70; 1.19)	0.91 (0.74; 1.11)	0.139
Ferritin (ng/mL)	941 (498; 1794)	875 (448; 1653)	1036 (610; 2095)	0.028
Procalcitonin (ng/mL)	0.30 (0.10; 0.76)	0.29 (0.09; 0.72)	0.30 (0.13; 0.78)	0.291
D-dimer (ng/mL)	1331 (702; 2872)	1510 (770; 3930)	1000 (592; 1930)	0.004
Leucocytes × 10^3^	8.48 (6.10; 12.08)	8.80 (6.36; 11.79)	8.18 (5.88; 12.41)	0.428
D-dimer, 2nd determination	1660 (937; 4429)	1993 (1028; 4729)	1306 (828; 3474)	0.028
D-dimer, last determination	1800 (991; 4750)	2257 (1088; 5036)	1480 (907; 3824)	0.029
Antibiotics (others)	229 (91.6)	128 (90.1)	101 (93.5)	0.340
Remdesivir	43 (21.9)	31 (20.1)	12 (28.6)	0.241
Anticoagulation	168 (71.5)	64 (50.4)	104 (96.3)	<0.001
LMWH	142 (67.6)	53 (49.1)	89 (87.2)	<0.001
Prone position	132 (50.2)	93 (59.2)	39 (36.8)	<0.001
Mechanical ventilation				0.907
≤15 days	141 (53.2)	84 (53.5)	57 (52.8)	
>15 days	124 (46.8)	73 (46.5)	51 (47.2)	
Central venous catheter				<0.001
None	50 (18.9)	4 (2.5)	46 (42.6)	
≤18 days	112 (42.3)	83 (52.9)	29 (26.9)	
>18 days	103 (38.9)	70 (44.6)	33 (30.6)	
Corticosteroids				<0.001
None	37 (14.0)	34 (21.7)	3 (2.8)	
<9 days	87 (32.8)	44 (28.0)	43 (39.8)	
≥9 days	141 (53.2)	79 (50.3)	62 (57.4)	

Data are means ± SD, frequencies (%), and medians (IQR). SDD: selective digestive decontamination; ICU: intensive care unit; LMWH: low-molecular-weight heparin.

**Table 2:** Incidence of infections according to SDD regimen.

Infection Exposure Data	Non-SDD N = 157	SDD N = 108	*p*-Value	Rate Ratio (95% CI)
Mechanical ventilation days	6354	6878	<0.001	0.204 (0.112; 0.371)
VAP	59	13		
Events per 1000 days	9.3	1.9		
Central venous catheter days	6375	8062	0.728	1.107 (0.624; 1.965)
Catheter bacteremia	20	28		
Events per 1000 days	3.1	3.5		
ICU days	9205	8724	0.087	0.406 (0.145; 1.138)
Secondary bacteremia	13	5		
Events per 1000 days	1.41	0.57		
ICU days	9205	8724	0.006	0.251 (0.095; 0.666)
Multi-resistant germs	21	5		
Events per 1000 days	2.28	0.57		

SDD: selective digestive decontamination; CI: confidence interval; VAP: ventilator-associated pneumonia; ICU: intensive care unit. Events per 1000 exposure days were calculated as follows: 1000 × frequency of events/exposure days for the entire cohort; *p*-values and rate ratios were obtained from the Poisson model.

**Table 3:** Multiresistant germs according to the SDD regimen and by infection.

			SDD	
		Total	No	Yes
VAP	*Pseudomonas aeruginosa*	4	2	2
	*Stenotrophomonas maltophilia*	4	4	0
	*Escherichia coli*	3	3	0
	*Klebsiella pneumoniae*	2	1	1
	*Pseudomonas putida*	2	2	0
Catheter bacteremia	*Acinetobacter baunmannii*	1	0	1
	*Enterococcus faecalis*	1	1	0
	*Klebsiella* spp.	1	1	0
	*Pseudomonas aeruginosa*	1	0	1
	Coagulase-negative *Staphylococcus*	1	1	0
Secondary bacteremia	*Pseudomonas aeruginosa*	2	2	0
	*Klebsiella pneumoniae*	2	2	0
	*Escherichia coli*	1	1	0
	*Pseudomonas putida*	1	1	0
Total	*Pseudomonas aeruginosa*	7	4	3
	*Escherichia coli*	4	4	0
	*Klebsiella pneumoniae*	4	3	1
	*Stenotrophomonas maltophilia*	4	4	0
	*Pseudomonas putida*	3	3	0
	*Acinetobacter baunmannii*	1	0	1
	*Enterococcus faecalis*	1	1	0
	*Klebsiella* spp.	1	1	0
	Coagulase-negative *Staphylococcus*	1	1	0

SDD: selective digestive decontamination; VAP: ventilator-associated pneumonia.

**Table 4:** Multivariate logistic regression for death.

Variables	*p*-Value *	AIC **	Odds Ratio (95% CI)
Age (per year)	0.003	291.5	1.043 (1.013; 1.073)
Apache II score on admission (per unit)	0.078	285.6	1.044 (1.995; 1.096)
Ln-last D-Dimer (per log unit) †	0.007	289.8	1.390 (1.090; 1.771)

Variables were selected using the best subset regression with the Akaike information criteria (AIC); CI: confidence interval. (*) Likelihood ratio test. (**) AIC value if the factor was removed from the model (AIC is a measure of lack of fitness). The AIC for the full model was 284.5; thus, if a factor was removed from the model, the resulting model was worse according to the AIC. (†) D-dimer values were logarithmically transformed to reduce skewness.

## Data Availability

Some of the data will be shared upon request.

## Supplementary Materials

The following supporting information can be downloaded at https://www.mdpi.com/article/10.3390/antibiotics11081016/s1. Table S1: MDR pathogen susceptibilities.
