# Supplementary material for: COVID-19 Secondary Infections in ICU Patients and Prevention Control Measures: A Preliminary Prospective Multicenter Study
Source: Antibiotics (Basel). 2022 Jul 28;11(8):1016. doi: 10.3390/antibiotics11081016 (PMC9405068; doi:10.3390/antibiotics11081016)
Supplement: Supplementary file 1 [file antibiotics-11-01016-s001.zip › antibiotics-1829345-supplementary.pdf]

Table S1. Multidrug resistant (MDR) pathogen susceptibilities

SDD	Germ	Colistin	Tobramycin	Gentamicin	Cefotaxime	Piperacillin Tazobactam	Amikacin	Cloxacillin	Ceftazidime	Cefepime	Aztreonam	Meropenem	Imipenem
No	*Klebsiella pneumoniae*	S (≤2)	R (>4)	R (>4)	R (>32)	R (>16)	S (≤8)			R (>8)	R (>4)	S (2)	S (4)
No	*Escherichia coli*		S (≤2)	S (≤2)	R (>32)		S (≤8)					S (≤0.12)	S (≤0.12)
No	*Escherichia coli*		S (≤2)	S (≤2)	R (>32)	S (≤8)	S (≤8)			R (>8)	R (>4)	S (≤ 0.12)	S (≤0.12)
No	*Escherichia coli*	S	S (≤2)	S (≤2)		S	S (≤8)		R (>32)	R (>8)		S (2)	S (≤1)
No	*Stenotrophomonas maltophilia*		R (>4)	R (>4)		R (>16)	R (>16)				R (>4)	R (>32)	
No	*Pseudomonas aeruginosa*		S (≤2)			S (16)	S (≤8)		S (4)		R (>24)	R (16)	R (>8)
No	*Pseudomonas aeruginosa*		S (≤2)			S (16)	S (≤8)		S (4)	S		R (16)	R (>8)
No	Coagulase negative *Staphylococcus*			R (>4)				R (>2)					
No	*Pseudomonas aeruginosa*		S (≤2)			S (≤8)	S (≤8)		S (≤1)		S (4)	S (1)	S (2)
No	*Pseudomonas aeruginosa*		S (≤2)			S (≤8)	S (≤8)		S (≤1)		S (4)	S (2)	R (8)
No	*Pseudomona putida*	R (≥16)	R (4)	R (2)		R	R (32)		R	R (16)	R	R	R (12)
No	*Klebsiella spp.*	R (≥16)	S (≤1)	S (≤1)		R	S (≤1)		R	R (16)	S (16)	R	R (12)
No	*Enterococcus faecalis*			R (SYN-R)			R (32)						S (2)
No	*Stenotrophomonas maltophilia*	S (≤0,5)	S (≤1)	R (2)			R (4)		R (2)	R (16)	R (≥64)	I (4)	R (≥16)
No	*Stenotrophomonas maltophilia*	S (≤0,5)	S (≤1)	R (2)					R (2)	R (16)	R (≥64)	I (4)	R (≥16)
No	*Klebsiella pneumoniae*	S (≤0,5)	S (≤1)	S (≤1)	I (≤0,25)	R (≥128)	S (≤1)		R (4)	I (0,25)	I (≤1)	S (≤0,25)	S (≤0,25)
No	*Stenotrophomonas maltophilia*	R (≥16)	R (≥1)	R (4)			R (4)		R (1)	R (16)	R (≥64)	R (≥16)	R (≥16)
No	*Escherichia coli*		R (≥16)	S (≤1)	R (≥64)				R (32)	R (16)			S (≤0,25)
No	*Pseudomonas putida*	R (≥16)	R (4)	R (2)		R	R (32)		R	R (16)	R	R	R (12)
No	*Pseudomonas putida*	R (≥16)	R (4)	R (2)		R	R (32)		R	R (16)	R	R	R (12)
No	*Klebsiella pneumoniae*	S (≤0,5)	R (≥16)	R (≥16)	R (≥64)	R (≥128)	R (32)		R (≥64)	R (≥32)	R (≥64)	R (12)	R (12)
Yes	*Pseudomonas aeruginosa*	S (1)	S	S		R	S		R	S	R	R	S
Yes	*Klebsiella pneumoniae*		R (4)	R (≥16)	R (≥64)	R (32)	R (≥64)		R (32)	R (≥32)		S (≤0.25)	
Yes	*Pseudomonas aeruginosa*		S(≤1)	S(≤1)		R (≥128)	S (0.5)		R (16)	R (8)	R (16)	S (2)	S (2)
Yes	*Pseudomonas aeruginosa*		S(≤1)	S(≤1)		R (≥128)	S (0.5)		R (16)	R (8)	R (32)	S (2)	S (2)
Yes	*Acinetobacterbaunmanii*	S(2)	S(≤1)	S(≤1)			S(≤2)					R(≥32)	R (16)

Table S1. (continuation). Multidrug resistant(MDR) pathogen susceptibilities

SDD	Germ	Ceftolozane Tazobactam	Ceftaz. Avibactam	Cefide-rocol	Levofloxacin	Ciprofloxacin	Ampicillin	Amoxicillin-clavulanate	Fosfomycin	Cotrimo-xazole	Carbapene-mase (+)	ESBL (+)
No	*Klebsiella pneumoniae*	R (>4)	S (≤2)			S (0.25)		R (>32)		R (>4/76)	Yes	Yes
No	*Escherichia coli*					R (>1)				R (>4/76)		Yes
No	*Escherichia coli*					R (>1)				R (>4/76)		Yes
No	*Escherichia coli*	S (≤1)	S (≤2)			R (>1)		R (≤8)	S (≤16)	R (>4/76)		Yes
No	*Stenotrophomonas maltophilia*				S (≤0.5)					S (≤2/38)		
No	*Pseudomonas aeruginosa*					R (>1)						
No	*Pseudomonas aeruginosa*					R (>1)						
No	Coagulase negative *Staphylococcus*				R(>1)		R (>4)	R (>2)				S (4/76)
No	*Pseudomonas aeruginosa*					S (0.25)						
No	*Pseudomonas aeruginosa*				S (≤0.5)	S (0.5)						
No	*Pseudomonas putida*					S (≤0,25)						Yes
No	*Klebsiella spp.*					S (≤0,25)						
No	*Enterococcus faecalis*				R (≥8)	R (≤8)	S (≤2)					
No	*Stenotrophomonas maltophilia*					I (1)				S (≥20)		
No	*Stenotrophomonas maltophilia*					I (1)				S (≥20)		
No	*Klebsiella pneumoniae*	S (0,5)				S (≤0,25)			S (≤16)	S (≥20)		Yes
No	*Stenotrophomonas maltophilia*					R (≥4)				S (≥20)		
No	*Escherichia coli*					R (≥4)	R (≥32)	R (≥32)	S (≤16)	R (≥320)		Yes
No	*Pseudomonas putida*					S (≤0,25)						
No	*Pseudomonas putida*					S (≤0,25)						
No	*Klebsiella pneumoniae*	R (≥32)	R (≥256)			R (≥4)			S (≤16)	R (≥320)	Yes	
Yes	*Pseudomonas aeruginosa*	S (2)	S (8)		S	S						
Yes	*Klebsiella pneumoniae*					R (≥4)	R (≥32)	R (≥32)	S (≤16)	S (≥320)		
Yes	*Pseudomonas aeruginosa*				R (≥8)	R (2)						
Yes	*Pseudomonas aeruginosa*				R (≥8)	R (2)						
Yes	*Acinetobacerbaunmanii*				R(≥4)	R(≥4)				R(≥320)		

SDD: Selective Digestive Decontamination. S: susceptibility. R: resistance. SYN: Synergy. ESBL: extended-spectrum beta-lactamase. Ceftaz.: ceftazidime
